# Viscous shaping of the compliant cell nucleus

**DOI:** 10.1063/5.0071652

**Published:** 2022-01-04

**Authors:** Richard B. Dickinson, Aditya Katiyar, Christina R. Dubell, Tanmay P. Lele

**Affiliations:** 1 Department of Chemical Engineering, University of Florida, Gainesville, Florida 32611, USA; 2 Department of Biomedical Engineering, Texas A&M University, College Station, Texas 77843, USA; 3 Artie McFerrin Department of Chemical Engineering, Texas A&M University, College Station, Texas 77843, USA; 4 Department of Translational Medical Sciences, Texas A&M University, Houston, Texas 77843, USA

## Abstract

The cell nucleus is commonly considered to be a stiff organelle that mechanically resists changes in shape, and this resistance is thought to limit the ability of cells to migrate through pores or spread on surfaces. Generation of stresses on the cell nucleus during migration and nuclear response to these stresses is fundamental to cell migration and mechano-transduction. In this Perspective, we discuss our previous experimental and computational evidence that supports a dynamic model, in which the soft nucleus is irreversibly shaped by viscous stresses generated by the motion of cell boundaries and transmitted through the intervening cytoskeletal network. While the nucleus is commonly modeled as a stiff elastic body, we review how nuclear shape changes on the timescale of migration can be explained by simple geometric constraints of constant nuclear volume and constant surface area of the nuclear lamina. Because the lamina surface area is in excess of that of a sphere of the same volume, these constraints permit dynamic transitions between a wide range of shapes during spreading and migration. The excess surface area allows the nuclear shape changes to mirror those of the cell with little mechanical resistance. Thus, the nucleus can be easily shaped by the moving cell boundaries over a wide range of shape changes and only becomes stiff to more extreme deformations that would require the lamina to stretch or the volume to compress. This model explains how nuclei can easily flatten on surfaces during cell spreading or elongate as cells move through pores until the lamina smooths out and becomes tense.

## I. INTRODUCTION

Normal cells like fibroblasts and invasive cancer cells migrate through interstitial spaces in the tissue. The large nucleus presents a challenge during such migration to fit through narrow interstitial spaces.^1^ The cell deforms the nucleus in such a way that nuclear shape is approximately coordinated with the shape of the cell. For example, when cells are patterned into varying aspect ratios, elongated cells exhibit elongated nuclei.^2,3^ Furthermore, the degree of nuclear elongation scales with the degree of cell elongation.

Competing models have been proposed to explain the coordination of cell and nuclear shapes. In one model, the shape of the nucleus is the result of a balance between compressive stresses exerted by actomyosin stress fibers abutting the nucleus and elastic restoring stress of the deformed nuclear shape.^3–6^ In these models, differences in the nuclear shapes in cells with different degrees of elongation or spreading are typically explained by differences in the spatial distributions of the actomyosin stress fibers. We have previously proposed an alternative viscous coupling model for stress generation on the nucleus, whereby movement of the cell boundaries transmits viscous stress through the intervening cytomatrix to a highly compliant nucleus.^2,7,8^ Here, we review the experimental and computational evidence for the viscous coupling model and revisit our previously proposed theoretical model for dynamic stress generation.

## II. EXPERIMENTAL EVIDENCE FOR A DYNAMIC MODEL FOR NUCLEAR SHAPING

### A. The elastic deformation hypothesis

Early tensegrity models of the cytoskeleton and the nucleus modeled the nucleus as storing elastic energy in its shape.^9,10^ Consistent with this hypothesis, rounding of trypsinized cells results in rounded nuclei, which supports the concept that flattened nuclei store elastic energy in their shape and relax to a rounded shape upon release of the forces that keep them flattened. Mechanical force application with micropipette aspiration to nuclei^11–13^ to deform them and then removal of the force results in a relaxation of the deformation, clearly showing that the nucleus can store elastic energy in its shape under applied forces by probes. Along these lines, a mechanical model was proposed^14^ in which actomyosin stress fibers that run along the long axis of an elongated endothelial cell and about the nuclear surface compress and elongate the normally circular nucleus. The elongated, deformed nucleus balances this stress by storing energy in its deformed shape. Similarly, compressive stresses from the actomyosin tensed cortex or apical stress fibers on top of the nucleus in cultured cells (the so-called F-actin cap) have been proposed to flatten the nucleus.^4–6,15^ These models have the following common features:

(a) Mechanical stresses that deform the nucleus originate in the actomyosin cytoskeleton through myosin-based contraction.
(b) Compressive stresses are transmitted to the nucleus through physical contact between actomyosin stress fibers (and/or actomyosin cortex) and the nuclear surface to flatten it in cultured cells.
(c) A removal of the stress by removal of the actomyosin stress should restore the nucleus to its original undeformed shape.
(d) Nuclear shape is determined by the instantaneous geometrical distribution of the actomyosin network or fibers.

As we discuss below, our recent experiments argue against these types of models.

### B. Questions raised by observations of nuclear flattening in spreading fibroblasts

Our early views on nuclear shaping were formed by experiments on the dynamics of nuclear flattening in spreading fibroblasts.^7^ These experiments revealed the following key features of the nuclear flattening process:

(a) The nuclear shape changes roughly conformed to cell shape changes during spreading until the nucleus asymptotically approached a limiting flattened morphology beyond which nuclear shape became insensitive to further cell spreading.
(b) Flattening of the nuclear shape occurred early in the spreading process long before the cell cortex impinged on the nuclear surface and before apical actomyosin stress fibers appeared.
(c) Nuclear flattening occurred at constant nuclear volume.
(d) Actomyosin activity, microtubules, intermediate filaments, as well as the Linker of Nucleoskeleton and Cytoskeleton (LINC) complex were all dispensable for flattening, as long as perturbations to them did not impact the spreading process.

In a later paper, we reported that the cell geometry—specifically the shape of the vertical cross section of the cell—establishes the nuclear shape in human mammary epithelial cell monolayers. Again, myosin inhibition did not affect nuclear heights in the monolayer.^8^

Overall, these experiments suggested that the process of cell spreading was necessary and sufficient to cause nuclear flattening. Furthermore, the mechanism for flattening required F-actin assembly that drove cell spreading exclusively but not actomyosin forces flattening the nucleus. These results led us to hypothesize a simple mechanical model for establishing nuclear shapes in cells, in which transmission of mechanical stress from the moving boundary to the nuclear surface causes it to move in response. Such a model can intuitively explain how the nuclear shape mimics the cell shape during cell spreading even when the cell boundary is separated from the nuclear surface by the intervening cytoplasm. It also provides a simple explanation for the correlation between cell spreading and nuclear flattening as follows. As the cell spreads at constant volume, the apical cell boundary moves downward, and the lateral boundaries move outward. These motions transmit stress to shape the nucleus similarly, i.e., move the apical surface of the nucleus downward and the lateral surfaces outward. Consequently, the nucleus is rounded in round cells and flattened in spread cells.

To our surprise, nuclei flattened without any interaction of stress fibers or cortical actin with the nuclear surface and even when myosin was inhibited. This observation suggested that the nucleus can flatten at much lower stresses than previously thought. How could the nucleus, commonly considered a stiff elastic object, be deformed to such extreme flattened morphologies even in the absence of myosin activity?

Moreover, how are stresses transmitted through the cytoplasm, given that the cortex does not contact the nucleus during the flattening processes? Also, cells spread on time scales of many minutes where any elastic stresses in the cytoskeleton are expected to dissipate. This assumption would argue against the notion that nuclear shapes are established and sustained by elastic cytoskeletal stresses.

### C. The nucleus is soft to cellular stresses during spreading because of excess lamina area

A key finding in Ref. 7 was that over a range of cytoskeletal disruptions, the height of nuclei in spread cells followed a universal dependence on the cell spreading area until becoming asymptotically independent at the larger spreading area. This result indicated that the initially rounded nuclear shape easily conformed to the cell shape during spreading until a mechanical limit was reached that prevented further flattening. Furthermore, the nucleus flattened with constant volume. Contrary to these findings, our computational model that utilized experimentally measured mechanical properties of the lamina^16^ suggested that the lamina of a spherical nucleus should prevent its flattening at constant volume.^7^ This prediction is because a sphere can only flatten at constant volume by increasing its surface area, but the lamina should be highly resistant to significant stretching under typical cellular forces. To explain this discrepancy, we hypothesized that the lamina in rounded nuclei must have an area in excess of that of a sphere of the same volume to allow any significant shape changes. This hypothesis predicts that a rounded nucleus must store excess area in wrinkles and folds that must disappear upon flattening. We confirmed this hypothesis through the following experiments.

First, we observed that nuclei in rounded cells had surface undulations unlike the nuclei in flattened cells; we estimated an excess area in rounded nuclei of about 20%–40% for NIH 3T3 fibroblasts.^7^ Second, we reported that folds in the nuclear lamina indeed disappeared due to the process of cell spreading in MCF10A cells.^8^ Nuclear wrinkling in rounded cells on soft substrates and smooth nuclei in flat cells have also been reported by others.^17,18^ Also, isolated nuclei become stiffer after an initial threshold strain, consistent with the lamina becoming taut.^11,19^ Third, knockdown of lamin A/C increased the degree of nuclear flattening and cell spreading in NIH 3T3 fibroblasts and MCF10A cells.^7,8^

**FIG. 1.**
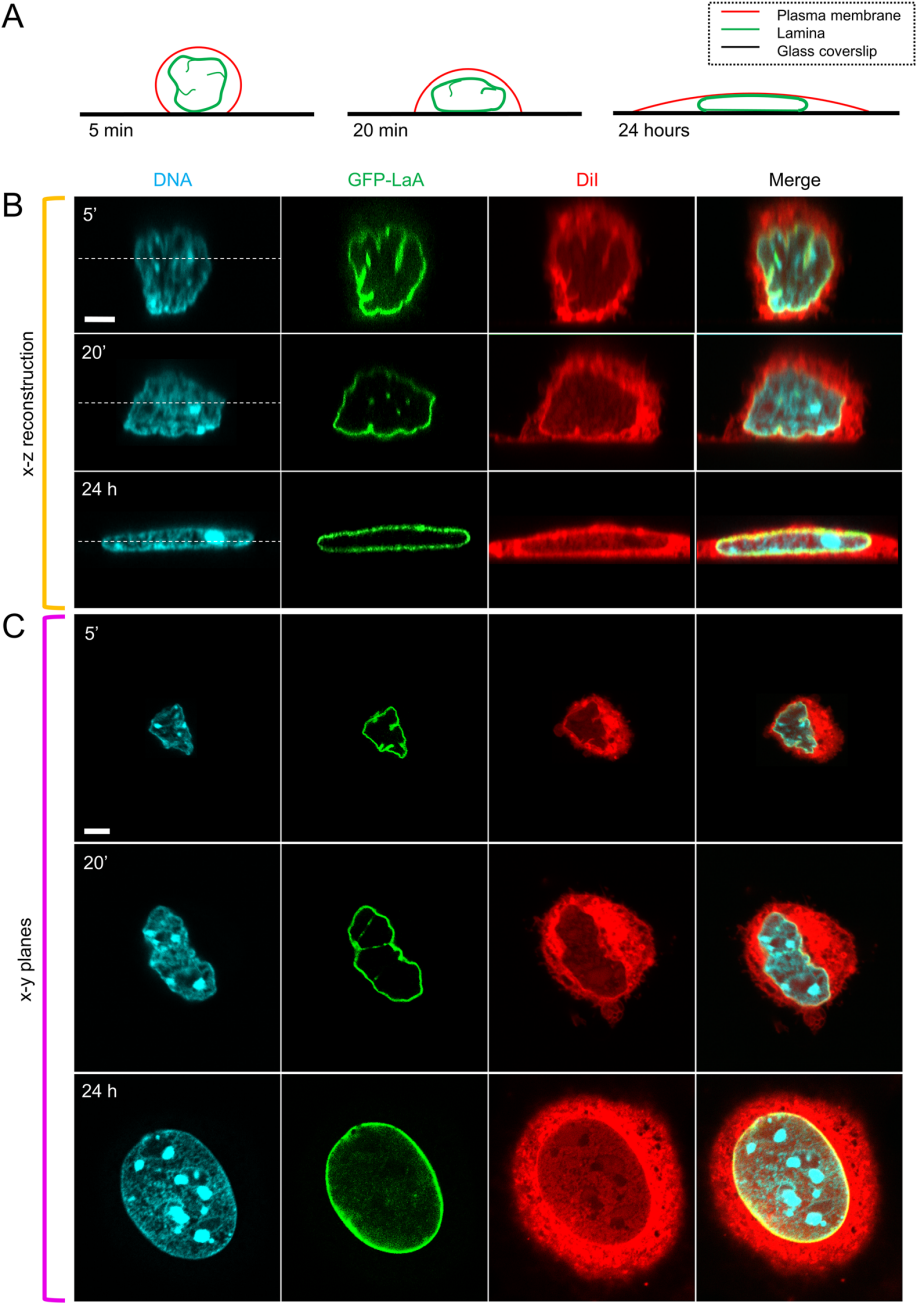
Cell spreading unfolds the excess area of the nucleus. (a) Schematic illustrates the process of cell spreading and the corresponding change in nuclear morphology. The nucleus in rounded cells is not spherical, and its lamina has excess surface area relative to that of a sphere with the same volume. The excess lamina area is stored in surface folds and undulations. (b) x–z reconstruction of NIH 3T3 fibroblasts stably expressing GFP-Lamin A/C (lamina, green), fixed and labeled with Hoechst H33342 (DNA, blue), and DiI D7756 (lipid dye, red), at different time points post-seeding: 5 min, 20 min, and 24 h (scale, 5 *μ*m), followed by (c) x–y views of their nuclear morphologies, respectively (scale, 5 *μ*m). The x–y views correspond to the plane marked by white dashed line in (b). As the cell spreads, the folds and undulations of the nuclear lamina ultimately disappear, at which point the stiff lamina prevents further spreading. Hence, spreading is geometrically limited by the amount of excess area.

**FIG. 2.**
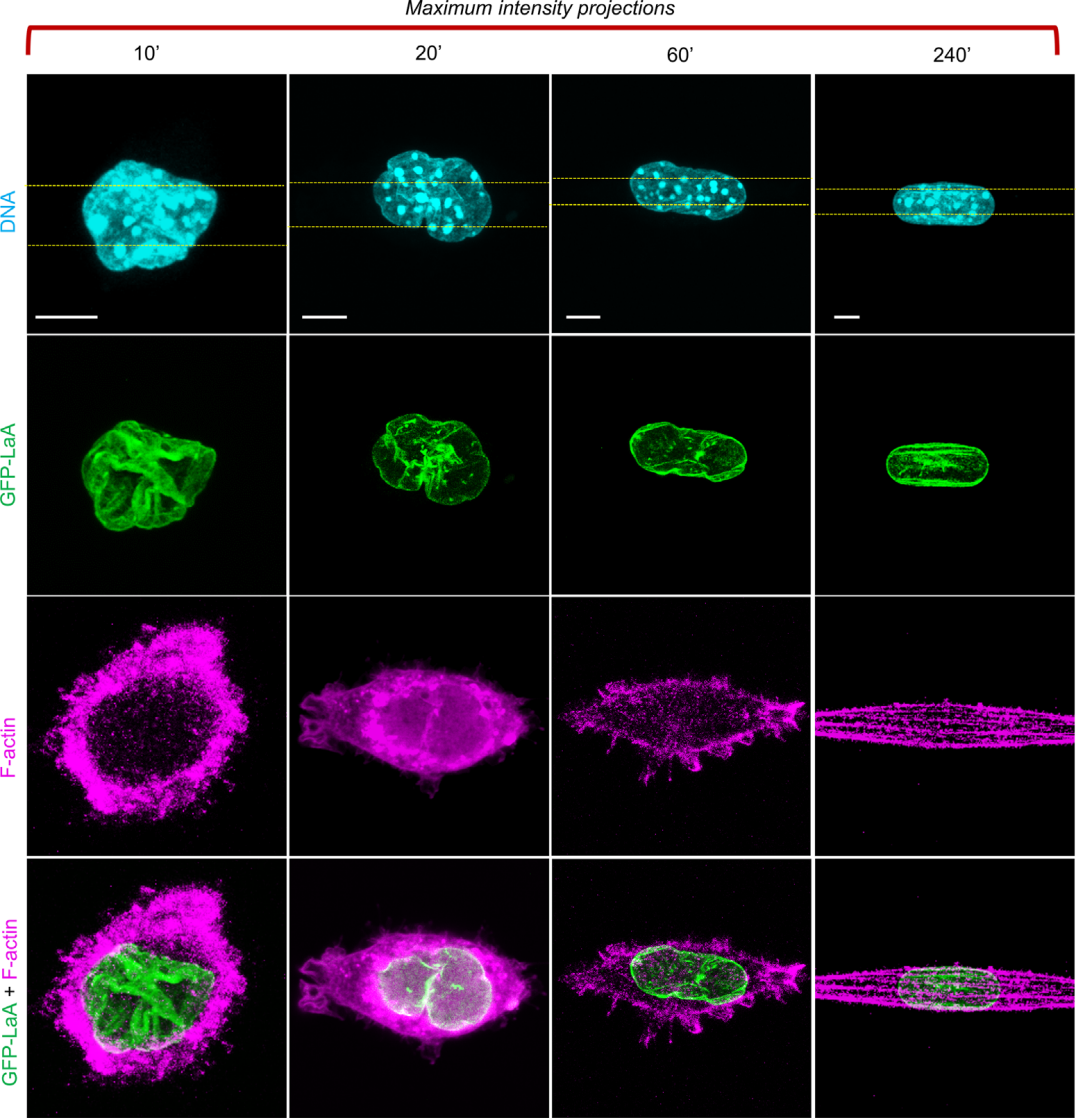
Elongating nuclei in cells spreading on a 5-*μ*m line pattern. Maximum intensity projections of NIH 3T3 fibroblasts stably expressing GFP-Lamin A/C (lamina, green) seeded on micro-contact printed 1D lines (represented with dashed yellow lines). The cells were fixed and labeled with Hoechst H33342 (DNA, blue), and Phalloidin (F-actin, magenta) at different time points post-seeding: 10, 20, 60, and 240 min (scale, 5 *μ*m). The nuclear shape changes mirror those of the spreading cells, because the nuclear surface is mechanically coupled to the cell boundary through the intervening cytoplasm. Eventually, the excess area stored in wrinkles and folds disappears, and the nucleus reaches a limiting pill-like shape. At later times, indentations into the lamina surface by stress fibers commonly appear (in the example, fixed at 240 min).

From these observations, a mechanical picture emerges of the nuclear shape being constrained geometrically by constant volume and constant lamina surface area during cell spreading. However, it is the excess surface area (relative to that of a sphere of the same volume) that allows reshaping of the nucleus across a wide range of possible morphologies with little mechanical work under these constraints. For example, the nucleus can easily transition from a rounded shape with folds and surface undulations as seen in round cells to flattened or elongated shapes seen in fully spread cells, as illustrated in Fig. 1 for cells spreading on a 2D surface and in Fig. 2 for cells spreading along a 1D line pattern. In both cases, the transition from a wrinkled lamina to a smooth lamina in the fully spread cells agrees with our hypothesized mechanical limit being reached when the lamina becomes tensed. Because these limiting morphologies are geometrically limited by the amount of excess surface, they can be determined without invoking other mechanical properties of the cell or nucleus. While these limiting shapes are approached before any appearance of stress fibers, at later times stress fibers do appear. On 1D lines, stress fibers align with the long axis of the cell, as shown in Fig. 2 (240 min), and commonly appear to indent the lamina surface. Given that the dorsal stress fibers are nearly linear, and the ventral stress fibers along the flat substratum below the nucleus are completely linear yet create similar indentations, we surmise it is unlikely that the stress fibers themselves are the source of the force that is indenting the nucleus. (By analogy, a tensed but straight rubber band generates no lateral force, only longitudinal tension.) Rather, any stress is likely transmitted from the cell boundary tension onto the nuclear lamina with the dorsal stress fibers intervening and the nucleus pressing down upon the ventral stress fibers on the substratum. Even so, our assertion that the nucleus is soft to shape changes at constant volume and constant lamina surface area would include shapes with surface indentations, provided the total lamina surface area and nuclear volume remain constant. Thus, the appearance of indentations does not in itself imply a significant stress on the nuclear surface.

Based on our observation that the nucleus flattened in a way that mimics the spreading cell shape in the absence of any source of static stress from the cytoskeleton, such as the cortex impinging on the nucleus or the presence of actomyosin stress fibers, we hypothesized that the nucleus is irreversibly shaped during flattening. This hypothesis implies that the nucleus does not store elastic energy that would cause it to recover its shape upon removal of external stress. We tested this hypothesis by removing the cell from around the nucleus of single elongated fibroblasts with micro-dissection.^3^ There was no shape relaxation of the elongated nucleus long after removal of the cell. Similarly, abnormally shaped breast cancer nuclei did not relax after removal of the cytoplasm. In a separate study, we used laser ablation to sever stress fibers that abut the nuclear surface in elongated fibroblasts. Again, laser ablation of stress fibers produced no expansion in the nuclear cross section.^20^ These findings confirm our hypothesis that the nucleus stores no elastic energy in its shape. Moreover, these findings are also consistent with our hypothesis that dissipative rather than static cellular stresses deform the nucleus.

### D. Moving cell boundaries drive cell shaping during migration

Any model for nuclear shaping must explain the observation that the nuclear shape closely conforms to the evolving cell shape during cell spreading by following the movement of the cell boundary. Furthermore, it must explain how stress develops on the nucleus despite no direct contact between the cell boundary and the nuclear surface and no visible stress fibers in the intervening space. Finally, the model must explain how the nuclear shape is preserved when the surrounding cytoplasm is removed and how the nucleus is shaped despite the lack of actomyosin activity. We hypothesized viscous coupling between the cell boundary and the nuclear surface through the intervening cytoplasm. In this model, other static stresses, such as actomyosin network tension, hydrostatic pressure, and internal tension of the nucleus interior, are assumed to be in balance with each other across the nuclear surface. Thus, they generate no net stress for shaping the nucleus.

The viscous coupling hypothesis predicts that the nuclear surface should move in the same direction as the proximal cell boundary in spreading and migrating cells. Likewise, the motion of the nucleus should abruptly stop when the cell boundary stops moving, as the viscous stress becomes zero on the nuclear surface. We tested both these predictions by tracking the motion of fibroblasts as they moved from 1D to 2D fibronectin patterns,^2,21^ as they underwent a shape change from an elongated morphology to a wider morphology. We observed that nuclear widening occurred from elongated to circular shapes only when the cell width expanded laterally in the region adjacent to the nuclear surface. Nuclear width remained unchanged when the cell width expanded in the front portion of the cell. Furthermore, movements of nuclear boundaries occurred in the same direction as that of the adjacent cell boundaries.^2^ Changes in cell shape produced changes in nuclear shape only when a cell boundary adjacent to the nuclear surface moved perpendicularly to the nuclear surface. We reported similar behavior during fibroblast migration in collagen gels.^2,21^ In other experiments, we found that local F-actin-rich reversible cell protrusions formed proximal to the nuclear surface in elongated fibroblasts reversibly widened the nucleus in the direction of the protrusion without any discernible motion of stress fibers.^20^ Overall, these experiments confirmed the first prediction—that the nuclear surface should move in the same direction as the proximal boundary.

In separate experiments, we tracked the elongational spreading of single fibroblasts on 5 *μ*m-wide micropatterned fibronectin lines of controlled lengths.^2^ On micropatterned lines that were sufficiently long, nuclear elongation ultimately stopped even as the cell continued to elongate in either direction, supporting the existence of a mechanical limit to nuclear elongation, similar to the mechanical limit on nuclear flattening. On short lines where cells were forced to spread less, nuclear elongation stopped abruptly when the cell boundary reached the edge of the line and ceased to move. Collectively, these observations strongly suggest that local motion of the cell boundary is required for nuclear shaping, which occurs through the deformation of local nuclear surfaces in the direction of nearby moving cell boundaries. In another study, we reported the existence of vertically upward cell membrane protrusions in spread breast cancer cells, which preceded the formation of apical nuclear deformation.^22^ Apical nuclear deformations correlated with the presence or absence of the apical cell membrane protrusions, again supporting the viscous coupling hypothesis. Consistent with our observations, others have suggested that the perinuclear, Arp2/3-dependent F-actin network exerts a lateral compressive force on the nucleus to elongate the dendritic cell nucleus as it migrates through narrow constrictions.^48^

Our dynamic model of nuclear shaping suggests that the nuclear shape in the cell is generated irreversibly by movements of the cell boundary. That is, at any instant, the nuclear shape is a cumulative result of incremental changes to its shape, caused by prior incremental changes in the shape of the cell, rather than the consequence of a static stress balance between cytoplasmic and nuclear forces. Thus, the history of how the cell changes shape is important. Furthermore, excess surface area permits a wide range of nuclear shapes at constant surface area and constant volume, allowing the nuclear shape to mimic the cell shape with little mechanical resistance until limiting shapes characterized by a tensed lamina are reached. For example, this model can easily explain the complex hour-glass shapes observed during migration through narrow interstitial spaces in tissue,^23,24^ which cannot be easily explained by actomyosin stress fiber-based compression.

In summary, the viscous coupling model makes the following testable predictions:

(a) Removal of cytoskeletal forces from the nuclear surface will not cause a relaxation of the elongated nuclear shape to a circular shape.
(b) Nuclear shape changes in cells during cell spreading occur at constant volume and constant area.
(c) Nuclear flattening does not require actomyosin activity.
(d) Flattened nuclei contain few or no folds or wrinkles in the nuclear lamina, while rounded nuclei contain them.
(e) During cell spreading, the nucleus stops responding to changes in cell shape after the folds in the nuclear lamina are removed.
(f) Resistance to nuclear compression is larger for nuclei lacking folds and undulations in the lamina.
(g) The nuclear shape changes only when changes in cell shape occur.
(h) The nucleus deforms at a rate proportional to the speed of motion of the cell membrane.
(i) The nuclear shape mirrors cell shape in cultured cells, both in x–z and x–y planes; for example, nuclear flattening correlates with cell spreading, or hour-glass nuclear shapes in cells migrating through confining channels mirror hour-glass shapes of cells.
(j) Rounding of nuclei in trypsinized cells occurs due to compressive stresses on the nuclear surface due to the retracting cell boundary.
(k) The nucleus is much softer to deformation in cells than the stiffness measured by mechanical probes like AFM or micropipette aspiration.

## III. CAVEATS FOR APPLICABILITY OF THE MODEL

### A. Nuclear volume is not always constant in cells

While nuclear flattening occurs at constant volume^7^ and constant area, and nuclear volume is constant during nuclear deformations in wild type (WT) and lamin A/C deficient fibroblasts as they squeeze through narrow pores,^24^ we have reported elsewhere that the nuclear volume increases by about ∼20% when cells migrate from 1D lines to 2D fibronectin patterns over time scales of several hours.^21^ Nuclear volume does change during the cell cycle and scales with cell volume,^25–29^ suggesting that the nuclear volume and cell volume are osmotically coupled. We have reported that the cell volume decreases upon myosin inhibition and scales with corresponding decreases in nuclear volume.^21^ Also, nuclear volume is different depending upon the dimensionality of culture^21^ and sensitivity to cell shape.^30^ A scaling between nuclear volume and cell volume has also been demonstrated by others.^25^ In summary, while physiological changes in the cell volume can certainly change nuclear volume, and while it may also be possible for cellular stresses to change nuclear volume, a key feature of our model is that excess lamina area allows extensive shape changes without requiring a change in the nuclear volume.

### B. Nuclear shape need not always mirror cell shape

The viscous coupling model stipulates that cellular shape changes drive nuclear shape changes. This does not imply that static nuclear shapes must always mirror cell shapes. During cell spreading, the shape of the nucleus stops responding to changes in cell shape after the excess area in the nuclear shape is completely unfolded, as we showed in Ref. 7. If the nuclear lamina becomes completely unfolded early in the cell spreading process, then the nucleus will reach a steady-state shape long before the cell takes on its own irregular shape. In this case, the nuclear shape will not closely reflect the final irregular cell shape. For example, when a cell is micropatterned on polygonal matrix patterns, the nucleus will not take on a polygonal shape. Moreover, the nucleus will not take on shapes with corners owing to the tension in the nuclear lamina that exists when the lamina is fully unfolded in spread cells.

There are other reasons for the shapes taken on by nuclei in static cells, such as indentations caused by the centrosome, creating nuclear kidney bean shapes. Also, in abnormal cells like tumor cells, there may be heterogeneous lamin distributions, which may result in asymmetrical shapes of the nucleus at a steady state.

## IV. VISCOUS STRESS TRANSMISSION THROUGH A CONTRACTILE NETWORK

### A. Stress transmission to the nucleus by movements of the cell boundary

How might viscous transmission occur from the cell boundary to the nuclear surface? Images of the cytoskeleton have shown that the intervening volume between cell and nuclear boundaries is spanned by a three-dimensional actomyosin network, showing a distinct punctate pattern of myosin II clusters.^31–33^ We, therefore, hypothesized that the viscous stress is transmitted between the cell boundary and nuclear surface through this three-dimensional contractile actomyosin network that links the nucleus to the cell boundary.

Computational models commonly treat the cytoplasm as a biphasic material with a contractile network phase—actomyosin cytoskeleton, interconnected organelles, etc.—and interstitial fluid phase—cytosol.^34,35^ Contractile stress in the three-dimensional space between the nucleus and the cell boundary arises from clusters of non-muscle myosin molecules pulling on the surrounding network of interconnected actin filaments.^31,32,36,37^ Various constitutive models have been proposed for the viscoelastic properties of the cytoskeletal network.^38^ On the timescale of cell motility, typically many minutes, the cytoplasm is viscous with a measured viscosity up to 10^6^-fold greater than that of water.^39,40^ However, measurements of viscosity can vary widely and depend on the method and the length and time scales being probed with measurements of whole-cell viscosity typically yielding larger values of 10 Pa s or more.^40,41^ On the longer time of cell migration, elastic energy stored in the network dissipates due to relatively slow filament turnover and cycles of binding and dissociation of molecular motors and other protein cross-linkages. Straining the network temporarily stores strain energy in the elastic filaments and linkages, but this energy dissipates upon dissociation of the linkages or upon filament turnover; and this dissipation of energy leads to viscous behavior on the longer length scales.

**FIG. 3.**
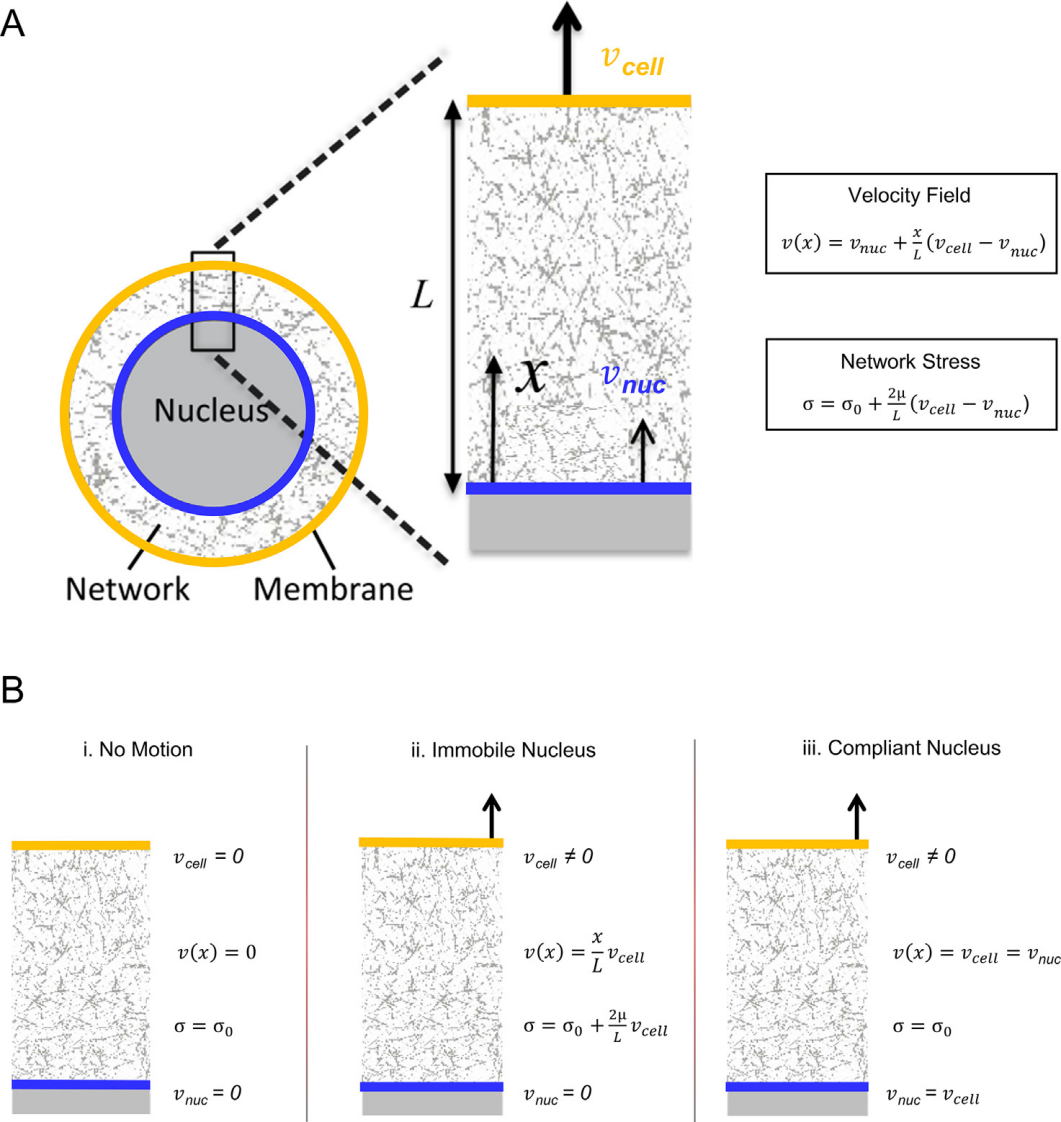
(a) Illustration of the stress transmission by the expansion or compression of a contractile network. (b) Applying a net stress σ to the surface (e.g., by changing hydrostatic pressure) that is greater than (or less than) the background network tension $\sigma_{0}$ will cause an expansion (or compression) of the network ${(v_{\mathrm{cell}}\neq v}_{\mathrm{nuc}})$. Conversely, expansion or compression of the network will generate uniform stress that is different from the background network tension.

We previously showed^7^ that the dynamics of nuclear shaping during cell spreading are captured well with a simple viscous model relating the stress to the rate of strain of the network phase of the cytoplasm connecting the nucleus to the cell boundary. In this model, pressure gradients within the interpenetrating liquid phase are assumed negligible on the slow timescale of cell migration, and the local concentration of components in the network phase is assumed constant due to sufficiently rapid network turnover (polymerization/depolymerization). To explain the model, we consider here the special case of unidirectional expansion or compression in the *x*-direction of the network phase spanning the gap between the nucleus surface and cell boundary (Fig. 3). In this case, the constitutive equation relating stress σ to rate of strain $\frac{{dv}_{x}}{dx}$ is

$$\sigma =\sigma_{0}+2\mu \frac{{dv}_{x}}{dx},$$ (1)

where $v_{x}$ is the network velocity. The first term in Eq. (1) accounts for the local contractile stress (of magnitude $\sigma_{0}$) in the network in the absence of any network motion, and the second term accounts for how stress depends on the local rate of strain; that is, expansion ( $\frac{{dv}_{x}}{dx}$ > 0) or compression ( $\frac{{dv}_{x}}{dx}$ < 0) of the tensed network correspondingly increases or decreases the network tension, σ, by an amount reflected by the viscosity parameter, μ. The stress balance requires $\frac{d\sigma }{dx}=0$ for this one-dimensional case implying a uniform tension throughout the network from the cell surface to the nuclear surface, given by

$$\sigma =\sigma_{0}+\frac{2\mu }{L}{(v_{\mathrm{cell}}-v}_{\mathrm{nuc}}),$$ (2)

and a velocity given by

$$v_{x}(x)=v_{\mathrm{nuc}}+\frac{x}{L}{(v_{\mathrm{cell}}-v}_{\mathrm{nuc}}),$$ (3)

where $v_{\mathrm{nuc}}$ is the velocity (in the x-direction) of the nuclear surface and $v_{\mathrm{cell}}$ is the network velocity at the cell boundary. (Generally, $v_{\mathrm{cell}}$ would be the difference between the velocity of the cell boundary and the retrograde flow velocity of the network being assembled at the cell boundary.)

The key insights of Eqs. (1)–(3) are illustrated in Fig. 3(b). First, when the surfaces do not move relative to each other ${(v_{\mathrm{cell}}=v}_{\mathrm{nuc}})$, the contractile network generates stress on the cell boundary and nuclear surfaces of magnitude $\sigma =\sigma_{0}$. Since the net stress on these surfaces must be zero, this cortical tension must be balanced by other stresses such as osmotic or hydrostatic pressure in the interpenetrating solution phase or by resisting the stress from the nucleus (e.g., pressure or tension in the nuclear interior due to its resistance to volume change, or surface tension effects when the nuclear surface is curved and tensed). Second, movement of the cell boundary relative to the nuclear surface ${(v_{\mathrm{cell}}\neq v}_{\mathrm{nuc}})$ increases or decreases the stress on the nuclear surface relative to the background tension, $\sigma_{0}$. Conversely, a change in stress on the boundaries (for example, due to a change in hydrostatic pressure or change in cortical tension) would cause the gap between the nucleus and the surface to expand or contract. If the nucleus is stationary, this change in stress must be balanced by a change in nuclear pressure and/or lamina tension. If, however, there is no mechanical resistance to movement of the nuclear surface (i.e., the nucleus is highly compliant), then $\sigma =\sigma_{0}$ and the nuclear surface would simply move at the same speed as the cell boundary ${(v_{\mathrm{cell}}=v}_{\mathrm{nuc}})$ regardless of the value of the viscosity, μ. Coupling the movements of the nuclear surface to the cell surface in this way would explain why the nuclear shape changes often mimic the cell shape changes even when the cell boundary and nuclear surface are separated.

## V. CONCLUSIONS

This Perspective offers a simple, parsimonious explanation for the coupling of cellular and nuclear shapes during cell motility based on excess nuclear surface area and viscous coupling between the cell and nuclear surfaces. First, the excess surface area of the nuclear lamina relative to that of a spherical nucleus of the same volume explains why the nucleus is highly compliant to shape changes (e.g., elongation or flattening), until the deformation is so extreme that it requires changes in nuclear volume or stretching of the actual lamina area. Consequently, the apparent mechanical properties of the nucleus should depend greatly on the extent to which the nucleus is deformed. That is, when the lamina is wrinkled, the nucleus is soft to deformation, and when the lamina is tensed, it is stiff to deformations that require stretching of the lamina or compression of the nuclear volume. Owing to its excess area, the nucleus can take a range of shapes with little resistance while maintaining constant volume and lamina area, even when the lamina is tensed. This simple geometric (rather than mechanical) model can explain the limiting shapes of nuclei in the spread or elongated cells as well as the resistance of nuclei moving through small pores without invoking any other mechanical properties of the cell or nucleus. Second, the surfaces of such a compliant nucleus will tend to track the cell boundary due to the mechanical coupling of the two surfaces through the intervening cytoplasm that is resistant to expansion or compression. Viscous stress transmission through the cytoplasm explains why nuclear shape changes mimic cell shape changes even without stress fibers and without the cell cortex impinging on the nuclear surface. Cytoplasmic mechanical resistance to deformation is assumed to be viscous on the timescale of minutes, consistent with measurements of cytoplasmic viscosity^39^ and with the observation that deformed nuclear shapes are preserved following removal of the cytoplasm.^2^ Finally, we offer a simple molecular model to explain how a contractile network can be viscous to expansion/compression/shear due to the action of clusters of myosin motors. While untested for three-dimensional contractile actomyosin networks, this model is an extension of our one-dimensional stress fiber model, which successfully explains the dynamics of contracting stress fibers.

While stress fibers, microtubules, or organelles are observed to locally indent the nuclear surface,^42^ we argue against these as driving the larger-scale nuclear shape changes (e.g., flattening or elongation). Stress fibers are far less abundant in migratory cells in 3D microenvironments compared to cells cultured on 2D surfaces. Furthermore, cancer cells regularly migrating in three-dimensional spaces feature highly elongated and irregularly shaped nuclei in highly dynamic and irregularly shaped cells.^1,23^ A stress fiber-based static model is highly unlikely to be able to explain the elongated, dynamic, and irregular morphologies of nuclei and the coordination of these nuclear shapes with the dynamic and irregular cellular morphologies. In contrast, our model can explain nuclear shaping in irregularly shaped, elongated cells easily, given that it is the motion of cell boundaries that generates the stress to shape the nucleus. Our understanding of the molecular mechanisms by which viscous transmission occurs through the intervening cytoplasm to the nuclear surface, particularly in myosin inhibited cells, remains incomplete. In addition to myosin II, we speculate that proteins, such as filamin, zyxin, and a-actinin, may play an important role in the transmission of stresses from the cytoplasm to the nuclear surface. Parsing the roles of these proteins in stress transmission is a key challenge for the future.

Beyond the impacts on apparent nuclear properties, the prediction that the lamina is tensed only in the spread or elongated cells may be physiologically important. For example, lamin A/C-stained mesenchymal stem cell nuclei display wrinkles^18^ when cultured on soft matrices (where cells spread less) but not on stiff matrices (where cells are more spread). Wrinkling correlates with YAP/TAZ (Yes-associated protein/transcriptional coactivator with PDZ-binding motif) localization in the nucleus suggesting a relation with gene expression.^18^ Also, stretching of the nuclear envelope during compression from rounded nuclei to flattened has been suggested to trigger signaling pathways that upregulate actomyosin contractility.^43^ Disrupting the LINC complex, which couples the nucleus to the cytoskeleton, has been shown to regulate genome-wide mRNA profiles^44^ and to disrupt endothelial mechanosensing.^45^ Such studies suggest that nuclear responses to mechanical stresses are likely to be important in a host of physiological contexts, including development and disease.

## VI. METHODS

### A. Cell culture

NIH 3T3 fibroblasts expressing GFP-Lamin A/C (a kind gift from Dr. Kyle Roux, Sanford Research, Sioux Falls, SD, USA) were cultured in Dulbecco's Modified Eagle's Medium with 4.5 g/L glucose (10–013-CV, Corning, Corning, NY, USA), supplemented with 10% v/v donor bovine serum (16030074, Gibco, Waltham, MA, USA) and 1% v/v penicillin/streptomycin (30–002-CI, Corning, Corning, NY, USA). Cells were maintained in a humidified incubator at 37 °C and 5% CO_2_.

### B. Fluorescent labeling

Cells were fixed in 4% paraformaldehyde (J61899, Alfa Aesar, Haverhill, MA, USA) at room temperature for 15 min and washed thrice with 1× PBS (21-040-CM, Corning, Corning, NY, USA). Hoechst H33342 (875756-97-1, Sigma-Aldrich, St. Louis, MO, USA) was used to stain DNA, DiI (D7756, Invitrogen, Carlsbad, CA, USA) was used to stain lipids, and Alexa Fluor-647 phalloidin (A22287, ThermoFisher Scientific, Waltham, MA, USA) was used to stain F-actin in fixed samples. Fixed samples were washed with 1× PBS thrice post-staining. All reagents were used at the concentration recommended by their respective manufacturers.

### C. Microscopy

Imaging was performed on an Olympus FV3000 (Olympus Scientific Solutions Americas Corp., Waltham, MA, USA) using Super-Apochromat 60× silicone oil immersion lens (UPLSAPO60XS2, Olympus Scientific Solutions Americas Corp., Waltham, MA, USA). A pinhole opening of 0.5 airy disk was selected and a z-step size of 130 nm to ensure overlapping z-stacks while sampling at less than half of the depth of focus (which corresponds to an optical section of 450 nm for 488 nm) to satisfy the Nyquist criterion and minimize photobleaching artifacts.^46^

### D. Micro-contact printing

35 mm hydrophilic polymer tissue culture dishes (80136, Ibidi, Martinsreid, Germany) were stamped with rhodamine-conjugated fibronectin (FNR01, Cytoskeleton, Inc., Denver, CO) in 1D lines 300 *μ*m in length and 5 *μ*m in width with by micro-contact printing as previously described^47^ for creating 1D fibronectin patterns. Briefly, a silicon wafer was etched with surface features using standard photolithography techniques. Then, polydimethylsiloxane, or PDMS (DC4019862, Sylgard 184 Silicone Elastometer Kit, Dow Corning, Midland, MI), was mixed at the manufacturer's recommended base to curing agent ratio of 10:1 (w/w) and cured at 60 °C in a convection oven for 4 h on the silicon wafer. The PDMS stamp was then peeled off and cut to the required shape and size. Rhodamine-conjugated fibronectin (FNR01, Cytoskeleton, Inc., Denver, CO) was diluted to a 20 *μ*g/ml concentration in DI water, and a 20 *μ*l drop was adsorbed onto the stamp surface for 30 min. Culture dishes were treated for 2 min with a low-frequency plasma cleaner unit (PE-25, PlasmaEtch, Inc., Carson City, NV) before printing them with the rinsed and dried rhodamine-conjugated fibronectin covered stamps. Non-printed regions of the dish were passivated with 200 *μ*g/ml PLL-g-PEG [poly(L-lysine)-g-poly(ethylene glycol)] solution (Surface Solutions AG, Dübendorf, Switzerland) overnight at 4 °C to prevent inadvertent protein adsorption and cell adhesion on non-printed regions.

## Data Availability

The data that support the findings of this study are available within the article.
